# Editorial: Drug discovery and nano delivery of natural products

**DOI:** 10.3389/fphar.2024.1402362

**Published:** 2024-04-10

**Authors:** Jun Ye

**Affiliations:** ^1^ State Key Laboratory of Bioactive Substance and Function of Natural Medicines, Institute of Materia Medica, Chinese Academy of Medical Sciences and Peking Union Medical College, Beijing, China; ^2^ Beijing Key Laboratory of Drug Delivery Technology and Novel Formulation, Institute of Materia Medica, Chinese Academy of Medical Sciences and Peking Union Medical College, Beijing, China

**Keywords:** drug discovery, nano delivery, natural products, nano-drug delivery systems, major diseases

Natural products refer to single-component or multi-component active agents that come from animals, plants, and other organisms and have a definite therapeutic effect. Natural products have the advantages of novel structure, high biological activity, favorable safety profile, and multiple pharmacological activities. They are an important source of new drugs and show great potential in the field of current drug research and development. However, many natural products are difficulty meeting the pharmacologic requirements due to low water solubility, poor stability, and poor oral bioavailability. Therefore, how to find natural products with therapeutic potential and use delivery technology to solve the problem of medicinal properties is very important.

Nano-drug delivery systems (NDDS) are composed of polymer materials and drug molecules assembled into nanoparticles with nanometer scale effects, which have multiple advantages over conventional delivery systems, such as enhanced drug solubility, targeted delivery to desired sites, sustained and controlled drug release, and improved transport efficiency. In this respect, NDDS have emerged as promising vehicles for improving the therapeutic index and reducing the toxicity of active pharmaceutical ingredients in recent years.

It is an advantageous strategy to use nano-drug delivery systems (NDDS) to deliver natural products. NDDS can not only significantly improve the solubility and stability of natural products but also improve the drug molecules across the membrane transport capacity, and improve the *in vivo* pharmacokinetic characteristics and tissue targeting, to improve the safety and efficacy of clinical treatment and compliance. With the increasing research and growing clinical applications of NDDS-based nanomedicine (such as Doxil^®^ and Abraxane^®^), NDDS has gained increasing attention in the field of drug research and development. However, due to the inherent physical and chemical properties of natural products, many natural products with good application prospects are difficult to be effectively encapsulated into nanocarriers due to poor lipid solubility and it is difficult to improve the biological characteristics of agents under the scale effect and surface properties of nanocarriers. Therefore, how to choose polymer materials to construct NDDS to improve the encapsulation efficiency and drug loading capacity is a key step to breaking through the technical barriers of nano-drug research and development.

This Research Topic published cutting-edge papers in the discovery, characterization, and nano delivery of natural products. Guo et al. explored the anti-colorectal cancer (CRC) effect of Erigeron breviscapus (Dengzhanxixin in China) injection (EBI) and elucidated its mechanism of action. The results showed that EBI inhibited the proliferation of CRC cell lines by activating the RIPK3/MLKL signaling pathway, and effectively suppressed the migration and invasion of SW620 cells. Ramulus Mori (Sangzhi) alkaloids (SZ-A) derived from twigs of mulberry was approved by the National Medical Products Administration in 2020 for the treatment of type 2 diabetes mellitus. Increasing evidence has confirmed that SZ-A exerts multiple pharmacological effects. Systematically investigated the pharmacokinetics and tissue distribution of SZ-A and found that SZ-A was rapidly and widely distributed in target tissues, with good metabolic stability and a low risk of triggering drug-drug interactions. Additionally, Liu et al. assessed the effect of SZ-A on gut microbiota in obese mice and explored the association among changes in gut microbiota, obesity, and lipid metabolism. The results showed that oral administration of SZ-A could significantly reduce body weight, fat mass, and the level of total cholesterol and low-density lipoprotein in serum in obese mice induced by a high-fat diet. Interestingly, SZ-A also regulated gut microbiota and changed the fecal metabolite composition of obese mice. Zhang et al. summarized the contemporary literature about local anesthetic adjuvants and highlighted their potential side effects while discussing the potential of exosomes as novel local anesthetic adjuvant drugs. Zhang et al. reviewed the latest research on nanomedicines based on natural products for the treatment of ulcerative colitis (UC) and their interactions with the gut microbiota, discussed the roles and functions of the gut microbiota and metabolites in UC and suggested challenges and future directions for improving the efficacy of nanomedicines for the treatment of UC. To overcome the poor water solubility and oral bioavailability of cryptotanshinone (CTS), Zhao et al. prepared CTS nanocrystals by precipitation and high-pressure homogenization using Poloxamer 407 as a stabilizer. The prepared nanocrystals significantly increased the saturation solubility and dissolution rate of CTS and improved its oral bioavailability in rats.

Through these published cutting-edge articles, we can more clearly recognize the great potential of natural products in the treatment of major diseases. At the same time, NDDS has great advantages in improving the inadequate physicochemical properties of natural products and overcoming multiple physiological barriers *in vivo*. Therefore, combining the remarkable therapeutic potential of natural products with the *in vivo* delivery advantages of NDDS is the future development direction of natural products, which will greatly enhance the drug-forming properties of natural products and promote the more rapid application of more natural products in the treatment of major diseases.

